# The determinants and longitudinal changes in vitamin D status in middle-age: a Northern Finland Birth Cohort 1966 study

**DOI:** 10.1007/s00394-021-02606-z

**Published:** 2021-06-17

**Authors:** Helmi Ikonen, Johanna Lumme, Jussi Seppälä, Paula Pesonen, Terhi Piltonen, Marjo-Riitta Järvelin, Karl-Heinz Herzig, Jouko Miettunen, Maarit Niinimäki, Saranya Palaniswamy, Sylvain Sebert, Marja Ojaniemi

**Affiliations:** 1 Center for Life-Course Health Research, Faculty of Medicine, University of Oulu, 90014 Oulu, Finland; 2 PEDEGO Research Unit, University of Oulu, 90014 Oulu, Finland; 3 Medical Research Center Oulu, Oulu University Hospital and University of Oulu, 90014 Oulu, Finland; 4 Department of Obstetrics and Gynecology, Oulu University Hospital, 90220 Oulu, Finland; 5 Department of Mental and Substance Use Disorders, South Carelia Social and Healthcare District, 53130 Lappeenranta, Finland; 6 Infrastructure for Population Studies, Faculty of Medicine, University of Oulu, 90014 Oulu, Finland; 7 Department of Epidemiology and Biostatistics, School of Public Health, Faculty of Medicine, St. Mary’s Campus, Imperial College London, London, W2 1PG UK; 8 Department of Life Sciences, College of Health and Life Sciences, Brunel University London, Kingston Lane, Uxbridge, UB8 3PH Middlesex UK; 9 Unit of Primary Care, Oulu University Hospital, 90220 Oulu, Finland; 10 Institute of Biomedicine, Medical Research Center, University of Oulu, 90014 Oulu, Finland; 11 Institute of Pediatrics, Department of Pediatric Gastroenterology and Metabolic Diseases, Poznan University of Medical Sciences, 60-572 Poznań, Poland; 12 Department of Pediatrics and Adolescence, Oulu University Hospital, 90220 Oulu, Finland; 13 Social Insurance Institute of Finland, 70 110 Kuopio, Finland

**Keywords:** 25-hydroxyvitamin D, 25(OH)D, Food fortification, Vitamin D fortification, Vitamin D supplementation, Population-based

## Abstract

**Purpose:**

Populations living in the Nordic countries are at high risk for vitamin D (VitD) deficiency or insufficiency. To reduce the risk, nationwide interventions based on food fortification and supplementation are being implemented. However, there is limited evidence about the impact of such public health campaigns on target populations.

**Methods:**

We studied an unselected sample of 3650 participants (56.2% females) from the longitudinal Northern Finland Birth Cohort 1966 with repeated measures of serum 25-hydroxyvitamin D [25(OH)D] at ages 31 (1997) and 46 (2012–2013). Timepoints corresponded to the period before and during the food fortification. We examined the effect of VitD intake from the diet and supplementation, body mass index and previous 25(OH)D concentration on 25(OH)D concentration at 46 years using a multivariable linear regression analysis. A 25(OH)D *z* score adjusted for sex, season, latitude and technical effect was used in the analysis.

**Results:**

We observed an increase of 10.6 nmol/L in 25(OH)D, when the baseline 25(OH)D was 54.3 nmol/L. The prevalence of serum 25(OH)D below < 50 nmol/L was halved. The changes were found for both sexes and were more pronounced in winter compared to summer months. Regular VitD supplementation had a significant positive effect on 25(OH)D at the age of 46, as well as had the dietary intake of fortified dairy products and fish, and the previous 25(OH)D concentration. However, the intake of fat-spreads albeit VitD-fortified, did not predict 25(OH)D.

**Conclusion:**

Our results demonstrated the positive impact of the fortification programme on VitD status in middle-aged population.

**Supplementary Information:**

The online version contains supplementary material available at 10.1007/s00394-021-02606-z.

## Introduction

In addition to the traditional role of vitamin D (VitD) in bone health, several studies have shown an inverse association between serum 25-hydroxyvitamin D [25(OH)D] and multiple non-skeletal medical conditions, including type 2 diabetes [1], cardiovascular diseases [2, 3], autoimmune diseases [4], certain cancers [5], depression [6], and all-cause mortality [7–9]. This might be important to acknowledge in the middle-aged population, where we observe steep increase in the prevalence of non-communicable diseases.

In the northern latitudes, where the daylight is reduced for a long winter period, the dietary intake of VitD-rich foods and oral supplementation are essential sources of VitD to prevent VitD deficiency [10, 11]. Unfortunately, only a few natural food products (fish, egg yolk, and certain wild mushrooms) contain significant amounts of VitD [12]. To ensure an adequate VitD status, assessed by serum 25(OH)D, among the population, some Nordic countries (Finland, Sweden, and Norway) have launched VitD food fortification programmes by adding VitD systematically to non-organic dairy products, fat spreads, breakfast cereals, and certain baby foods [13, 14].

In December 2002, the National Nutrition Council (NNC) of Finland has granted approval for (i) all fluid dairy products (excluding organic) and respective plant-based alternatives to be systematically fortified with 0.5 µg/100 g and (ii) all fat spreads, excluding butter, to be systematically fortified with 10 µg/100 g with vitamin D_3_ [15]. The first evaluation of this programme has shown that the prevalence of serum 25(OH)D concentrations below 50 nmol/L were still observed in 21.3% of the population [16], and the recommendation was, therefore, doubled in the second fortification wave in 2010 [15]. In addition to fortification, the NNC recommends individual oral supplementation with 10 µg/day of vitamin D_3_ during the darkest time of the year for adults not regularly consuming fish and food products fortified with VitD (i.e., dairy products or fat spreads) [17].

A study by Jääskeläinen et al*.* [18] reported an average improvement of 17 nmol/L in serum 25(OH)D concentrations [18]. The study samples were from the Finnish national health survey Health2000 and 2011–2012 (among participants over 30 years, *n* = 6134 in 2000 and 4051 in 2012, mean age = 56 years) conducted before and after the fortification waves [18]. However, studies from Sweden and Norway conflicted with this observation [19, 20]. They reported a relatively stable VitD status among the general population in recent decades despite the start of VitD fortification programmes (Online resource 1) [19, 20]. The Norwegian study also observed a moderate correlation between VitD status in 1994 and 2008, suggesting tracking of serum 25(OH)D and, thus, the stability of VitD status over the years [20].

There is a paucity of studies to address the possible independent effect of VitD fortification programmes on the VitD status in the general population taking into account a variety of additional risk factors, including amongst other BMI, VitD intake from other dietary sources and supplementation as well as previous VitD status. Therefore, the objective of the present study was to evaluate this question using longitudinal data in a population-based birth cohort from Northern Finland sampled before and during the national food fortification. We aimed at analyzing whether the effect of fortification was independent from the effect of supplementation. Our study population consists of adults followed from young adulthood until middle-age and is characterized by an increasing incidence of chronic diseases and females reaching menopausal age. We consider this group interesting in the light of VitD status and from a public health perspective, as representing a relevant target for preventive actions.

## Subjects and methods

### Study population

**Fig. 1:**
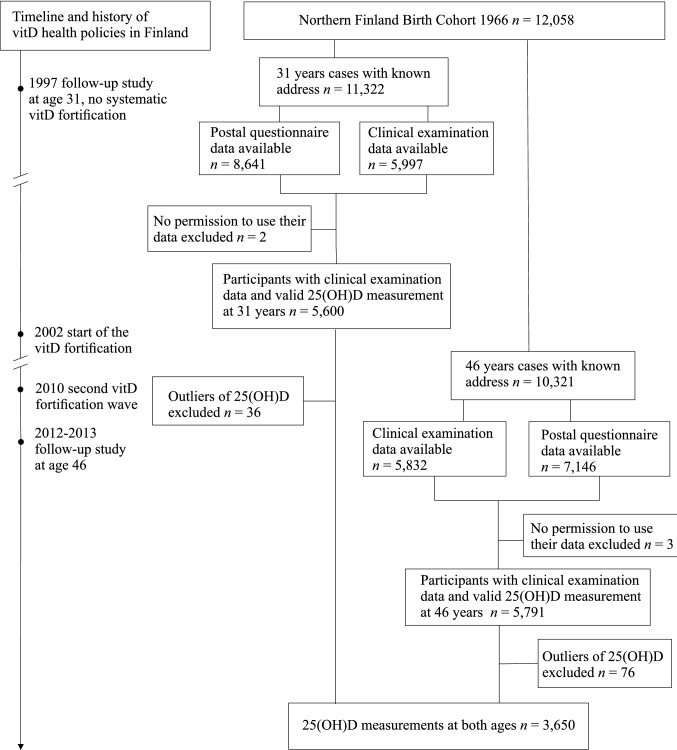
Flowchart of the study population (*n* = 3650) of the Northern Finland Birth Cohort 1966 at the 31-year (1997) and 46-year (2012–2013) follow-ups, including the timelines and history of the start of vitamin D fortification

The study population was derived from a prospective, general population-based birth cohort, the Northern Finland Birth Cohort 1966 (NFBC1966). This cohort has been described in detail elsewhere [21, 22], including two studies about VitD [23, 24]. In this study, we included the cohort participants who attended both the 31-year (1997) and 46-year (2012–2013) clinical follow-ups, before and during NNC launched food fortification programme (Fig. 1). At both timepoints, data from postal questionnaires and clinical examinations, including blood samples, were obtained.

The measure of serum 25(OH)D concentrations was available from a total of 5,571 participants (51.9% females) at 31 years and 5,659 (55.8% females) at 46 years (Fig. 1). We excluded the participants with the outlier values using the first quartile cutoff − 1.5 × IQR for the lower limit and the third quartile cutoff + 1.5 × IQR for the upper limit) [25]. A total of 112 measurements were considered outliers. At 31 years all 53 measurements were from the upper end. At 46 years, 75 out of 76 measurements were from the upper end (Online resource 2). In the analysis, we retained those participants with available 25(OH)D measurements at both timepoints (*n* = 3650). To identify possible selection bias, we examined the VitD status and the characteristics of the total population (i.e., VitD available at either timepoint) and the retained samples (i.e., VitD available at 31 years and 46 years). We found no differences in the vitD status or descriptive characteristics between full sample and those with the repeated measures (Online resource 3).

To define VitD status, we used serum 25(OH)D cutoffs of 30, 50, and 75 nmol/L for the VitD status groups based on both the IOM and ES guidelines [26, 27].

### Serum 25(OH)D measurements at 31 and 46 years

During the clinical examination visits at 31 and 46 years of age, blood samples, which were preceded by an overnight fast, were drawn between 8 and 11 am. The samples were frozen at − 70 °C until analyzed. At 31 years, the participants’ serum concentrations of 25(OH)D were determined using liquid chromatography–tandem mass spectrometry (LC–MS/MS; Elstree, Hertfordshire, UK). This detailed procedure has been described previously [23, 28]. A subset of the blood samples was later analysed with a chemiluminescence microparticle immunoassay (CMIA) Architect i2000SR automatic analyzer (Abbott Diagnostics). This assay has been certified according to the Center for Disease Control and Prevention’s Vitamin D Standardization-Certification Program [29]. An equation was calculated to convert the 31-year 25(OH)D concentrations to the Vitamin D Standardization Program-calibrated concentrations [30]. The calibrated concentrations were used in the final analyses. At age 46, the serum 25(OH)D concentrations were measured using the Architect i2000SR automatic analyzer (Abbott Diagnostics). The CV derived from the repeated quality-control samples included in the assay with the study samples were calculated. The CV for the internal control samples was ≤ 3.6% across the working range.

### Covariates

The possible covariates for the study were selected according to the literature in the field, our own testing and other specific factors related to the present study [23, 31]. For ages 31 and 46 years, we had information regarding the participants’ BMI, marital status, occupational and educational status, physical activity, smoking, alcohol consumption, season of blood sampling sample collection, and latitude of residence. In addition, for age 46 years, we had the information regarding VitD intake from diet and the use of VitD supplements.

During the clinical examinations at both timepoints, the participants’ height (cm) and weight (kg) while wearing light clothing were measured by well-trained nurses. BMI (kg/m^2^) was calculated using height and weight [32]. The information from the postal questionnaires was used when the measured weight and height were missing [33]. The self-reported and clinically measured BMI were verified as providing similar results [33].

From the postal questionnaires: Marital status was categorized as married (including married and cohabiting) and unmarried (not cohabiting/married, divorced, widowed). Occupational status was categorized as upper-level employees, lower-level employees/entrepreneurs, manual workers/farmers, and non-workers by current occupational status. Educational status was defined and categorized into three groups, namely, basic, secondary, and higher level, based on the information in the postal questionnaires. Physical activity was calculated based on the frequency and duration of leisure time activities [34] as metabolic equivalent of task (MET) scores in hours per week [34]. Smoking status was categorized as non-smokers, occasional/former smokers, and active smokers. Alcohol intake was calculated as g/day (d) based on the consumption of beer, wine, and spirits in 6 months prior to completing the questionnaire [35]. Alcohol intake was further categorized as abstainers (0 g/d), low-risk drinking (≤ 40 g/d for males and ≤ 20 g/d for females) and high-risk drinking (> 40 g/d for males and > 20 g/d for females) [36].

Season of blood sampling was defined based on the date of the participants’ clinical examinations at ages 31 and 46. The seasons were categorized as low VitD season (November–May) and high VitD season (June–October) [37]. At age 46, blood samples were obtained throughout the year, but at age 31, clinical examinations were not performed in February and March due to the holiday season in Finland.

Latitude was defined by the participants’ residence at the time of the follow-up studies in 1997 and 2012–2013. This information was collected from the Finnish population register center and categorized as 60° N (Helsinki and other provinces of middle and southern Finland), 65° N (the city of Oulu), and ≥ 65° N (other northernmost provinces of Oulu and Lapland) [23].

The participants’ dietary intake of VitD at the age of 46 was evaluated based on a food frequency questionnaire assessing frequency of food consumption during the preceding 6 months, as previously described in detail [38, 39]. Briefly, the National Food Composition Database in Finland, which is maintained by the National Institute for Health and Welfare [12], was used to evaluate VitD consumption from dairy products, spreadable fats, and fish.

- The consumption of dairy products was established from the question “How many glasses (0.2 L) do you usually drink/eat per day of: 1. Milk, 2. Sour milk, 3. Other dairy products (e.g., yoghurt, other fermented milk products, ice cream)?” All dairy products except cheese were estimated to contain 1 µg of VitD per 100 g [12, 17, 38, 40].
- The VitD content in fat spreads varies according to spread types: butter and organic butter contain 0 µg/g of VitD, vegetable oil spreads and plant-based sterol and stanol margarines 0.2 µg/g, and vegetable oil mixtures 0.1 µg/g. The consumption of fat spreads was established from the answers to the questions: “What type of bread spread do you usually use?” “How many times do you eat bread per day?” and “How much spread do you put on a slice of bread?”
- A serving of fish (approximately 150 g) was estimated to contain 13.4 µg of VitD based on information regarding the 10 most consumed fish from the Finnish National Food Composition Database [12]. If fish was eaten almost daily, the VitD dose was approximated to be 13.4 µg/d; if twice a week, 3.8 µg/d; and if once a week, 1.9 µg/d. Less than that was estimated to be 0 µg of VitD [12, 38].

We estimated the use of VitD supplements at the age of 46 using the following information from the postal questionnaires:

- The name of the product,
- The VitD content of the product informed by the participant, or if missing, by the product manufacturer,
- The number of tablets per day,
- The frequency of supplementation; the frequency of supplementation was estimated as “regular use” if the participant answered that they used VitD daily or regularly. All other answers (every other day, twice a week, during dark time, etc.) were considered “irregular.”

We included all VitD-containing supplements, including multivitamins, calcium, and omega-3 supplements. In cases where the VitD content was missing, it was estimated using the name of the product and the VitD content informed by the product manufacturer. The question regarding the use of vitamins, nutritional supplements, and medications was open-ended, missing information were classified as “non-users”. Participants who indicated the frequency of VitD supplementation were included even if the dose of the VitD supplement was missing (*n* = 76).

To control for the effect of different covariates on serum 25(OH)D, we standardised the measures of serum 25(OH)D as z-scores adjusting for sex, seasonal, geographical and technical variation (batch correction only for 31-year measurements). The z-scores were formed separately in the different combinations of the categories of the aforementioned variables. The resulting z-scores were combined into one variable, and these z-score variables for the 31- and 46-year 25(OH)D concentrations were used in the regression analysis.

### Statistical analyses

The normal distribution of the continuous variables was assessed visually using histograms with normality curves. To assess differences by VitD status groups and time, we used Pearson’s Chi-square test for categorical variables, one-way analysis of variance for normally distributed continuous variables and Kruskal–Wallis test for the non-parametric continuous variables. To study the differences in VitD intake characteristics between the sexes, we used an independent samples *t* test, Pearson’s Chi-squared test, and the Mann–Whitney *U* test.

To assess the effect of different covariates on 25(OH)D, we performed a multivariable linear regression analysis with 46-year 25(OH)D z-score as the dependent variable and the intakes of fluid dairy products, fat spreads and fish, supplement use, BMI and 31-year 25(OH)D *z* score as independent variables. We tested for marital status, physical activity, smoking, alcohol consumption, occupational and education variables and their relevant two-way interactions in the model, but no interaction was found, and their effects did not change the estimates of the other variables in the model. As a sensitivity analysis, we ran a similar regression analysis including the outlier data and an another with exclusion of females using oral contraceptives and hormonal replacement therapy (Online resource 2), since there is evidence for a difference in 25(OH)D among this group [23, 24].

**Fig. 2:**
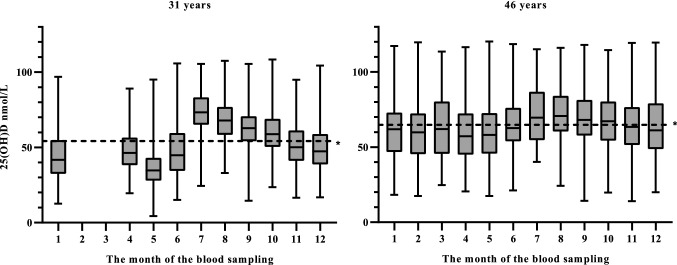
Monthly variations in serum 25(OH)D concentrations at ages 31 (1997) and 46 years (2012–2013) in the Northern Finland Birth Cohort 1966. *Dashed line showing the mean of all the 25(OH)D measurements at 31 and 46 years

**Fig. 3:**
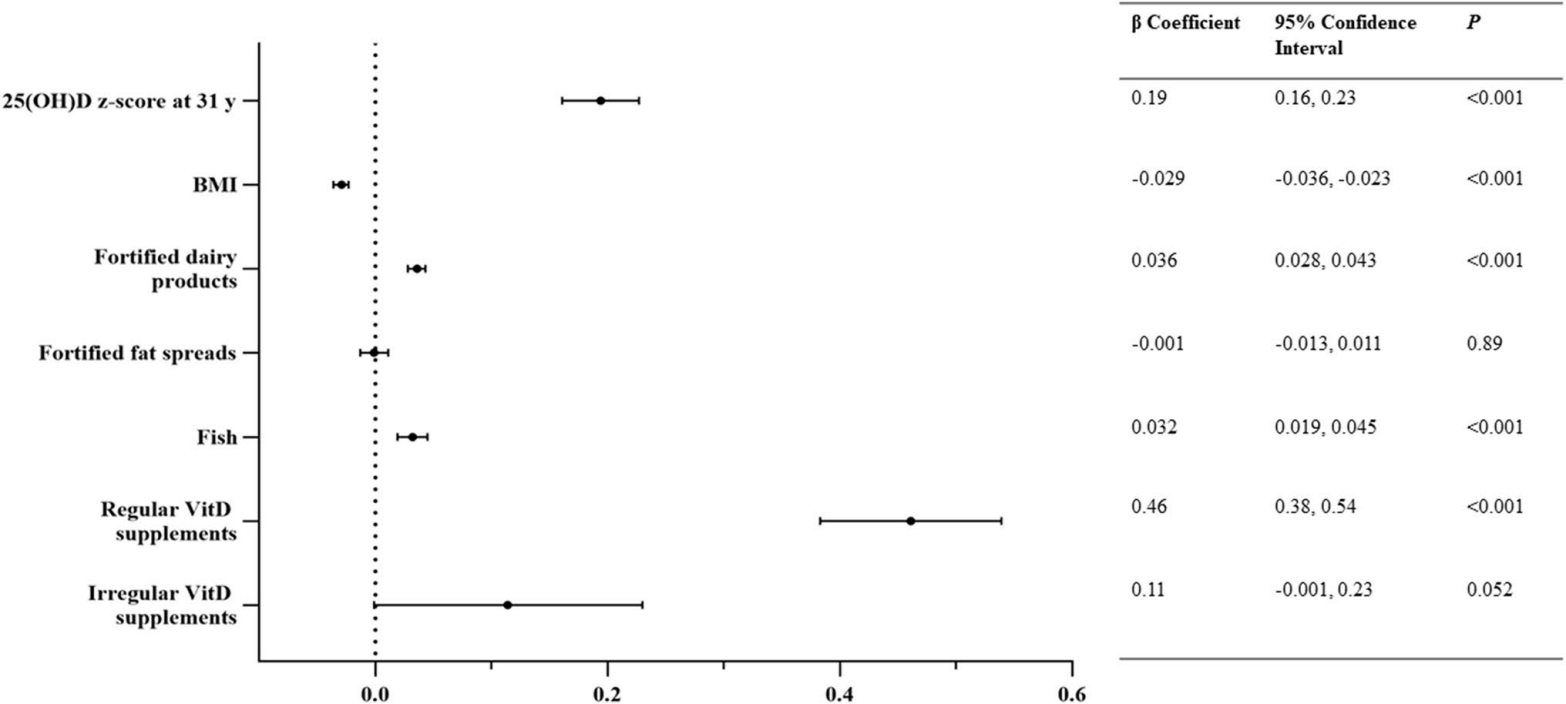
Results of the multivariable linear regression analyses of the 25(OH)D *z* score with different exposures at age 46 (2012–2013) in the Northern Finland Birth Cohort 1966. For VitD supplementation, no VitD supplementation was used as a reference category

The statistical analyses were performed using IBM SPSS Statistics for Windows, Version 25 (IBM Corp. Armonk, NY). Figure 1 was executed using CorelDRAW Graphics Suite 2019, Version 21.0.0.593 (Corel Corporation, Canada) and Figs. 2 and 3 using GraphPad Prism Version 8.0.1.244 (GraphPad Software, San Diego).

## Results

### VitD status in NFBC1966

The descriptive characteristics of the studied population at ages 31 (in 1997) and 46 (in 2012–2013) and the differences by VitD status group are shown in Table 1. BMI decreased and physical activity increased towards the highest VitD status group, and the magnitude of the difference between the lowest and the highest VitD status groups was larger at 46 years compared with 31 years. A higher proportion of measurements was taken during the high VitD season at 31 years, whereas it was the opposite at 46 years: a higher proportion of measurements were taken during the low VitD season.

**Table 1:** Descriptive characteristics and differences in the study population at 31 (1997) and 46 years (2012–2013) by 25(OH)D cutoffs in nmol/L

		31 years (*n* = 3541–3650)					46 years (*n* = 3462–3650)			
	< 30	30–50	50–75	> 75	*P*	< 30	30–50	50–75	> 75	*P*
Females^a^, % (*n*)	60.7 (210)	55.3 (671)	56.7 (897)	54.0 (276)	0.23	51.1 (47)	55.2 (422)	55.9 (972)	57.9 (610)	0.445
Marital status^a^, % (*n*)					0.97					0.016
Married	75.4 (258)	75.1 (903)	75.0 (1,176)	74.1 (375)		73.3 (63)	75.3 (545)	79.4 (1,319)	81.0 (825)	
Unmarried	24.6 (84)	24.9 (300)	25.0 (391)	25.9 (131)		26.7 (23)	24.7 (202)	20.6 (343)	19.0 (193)	
Occupational status^a^, % (*n*)					0.03					0.27
Higher level employee	18.2 (59)	21.8 (258)	17.0 (261)	16.7 (83)		10.7 (9)	18.2 (131)	17.9 (295)	18.2 (184)	
Lower level employee/entrepreneur	41.2 (134)	40.2 (475)	41.6 (640)	41.0 (204)		31.0 (26)	29.0 (209)	28.3 (467)	27.9 (282)	
Manual worker/farmer	30.2 (98)	27.1 (321)	27.4 (421)	28.1 (140)		52.4 (44)	46.0 (332)	49.6 (819)	48.8 (493)	
Not working	10.5 (34)	10.9 (129)	14.0 (215)	14.3 (71)		6.0 (5)	6.8 (49)	4.2 (70)	5.1 (52)	
BMI^b^, kg/m^2^	24.6 ± 4.2	24.5 ± 4.0	24.6 ± 4.2	23.9 ± 3.4	0.013	27.9 ± 5.4	28.2 ± 5.5	26.8 ± 4.7	25.8 ± 4.2	< 0.001
Physical activity^b,^^c^, MET hours/week, median (IQR)	10.2 (13.4)	10.9 (15.8)	11.9 (17.7)	12.9 (19.7)	0.007	10.1 (21.9)	11.9 (16.5)	13.1 (16.8)	15.6 (18.8)	< 0.001
Smoking^a^, % (*n*)					0.84					< 0.001
Non-smoker	49.1 (168)	50.3 (601)	47.4 (740)	49.5 (251)		43.0 (37)	53.9 (387)	55.4 (914)	54.9 (554)	
Former smoker	26.3 (90)	25.3 (302)	27.5 (430)	26.4 (134)		20.9 (18)	25.8 (185)	26.9 (444)	29.6 (299)	
Current smoker	24.6 (84)	24.5 (293)	25.1 (392)	24.1 (122)		36.0 (31)	20.3 (146)	17.6 (291)	15.5 (156)	
Alcohol consumption^a,d^, % (*n*)					0.015					< 0.001
Abstainer	11.9 (40)	11.4 (134)	9.2 (140)	6.2 (31)		12.8 (11)	12.0 (87)	10.9 (182)	8.3 (85)	
Low-risk drinker	83.8 (280)	84.5 (996)	85.6 (1,306)	87.8 (441)		68.6 (59)	78.4 (569)	82.1 (1,366)	85.0 (870)	
High-risk drinker	4.2 (14)	4.2 (49)	5.2 (80)	6.0 (30)		18.6 (16)	9.6 (70)	7.0 (116)	6.7 (69)	
Season of the blood sampling^a,e^, % (*n*)					< 0.001					< 0.001
High VitD season	24.9 (86)	39.5 (475)	77.5 (1,216)	89.0 (455)		13.0 (12)	26.3 (201)	44.7 (778)	51.4 (541)	
Low VitD season	75.0 (260)	60.5 (728)	22.5 (354)	11.0 (56)		86.2 (81)	73.7 (564)	55.3 (962)	48.6 (512)	
Latitude^a,f^, % (*n*)					< 0.001					< 0.001
60 °N	16.5 (57)	20.5 (249)	12.7 (201)	7.6 (39)		17.4 (16)	15.4 (118)	18.7 (325)	24.0 (253)	
65 °N	16.5 (57)	20.1 (244)	21.0 (332)	24.5 (125)		27.2 (25)	32.5 (249)	28.5 (496)	26.4 (278)	
≥ 65 °N	67.0 (232)	59.3 (719)	66.3 (1,048)	67.8 (346)		55.4 (51)	52.0 (398)	52.8 (919)	49.6 (522)	

Values are displayed as percentages with numbers in parentheses as % (*n*) or mean (SD), unless otherwise indicated

*MET* metabolic equivalent of task

^a^Differences between the groups were tested using Pearson’s Chi-squared test for categorical variables

^b^Differences between the groups were tested using one-way analysis of variance for normally distributed and the Kruskal–Wallis test for non-parametric continuous variables

^c^The MET physical activity scores in hours per week (frequency and duration of leisure time activities)

^d^Alcohol intake: abstainer (0 g/d), low-risk drinker (males ≤ 40 g/d, females ≤ 20 g/d), and high-risk drinker (males > 40 g/d, females > 20 g/d)

^e^High VitD season: summer (1 June–30 August) and autumn (1 September–31 October). Low vitamin D season: winter (1 November–31 March) and spring (1 April–31 May)

^f^Latitude: 60°N, Helsinki and surrounding areas; 65°N, the city of Oulu; > 65°N, the northernmost provinces of Oulu and Lapland

The VitD status of the studied population (Table 2) showed that, on average, serum 25(OH)D concentrations increased by 10.6 nmol/L (SD 24.4) during the follow-up. The prevalence of 25(OH)D < 50 nmol/L decreased nearly by half (42.7% at age 31 and 23.5% at age 46). In addition, 25(OH)D > 75 nmol/L doubled from 31 to 46 years. Those having serum 25(OH)D < 30 nmol/L at 31 years, had the highest increase in 25(OH)D in the follow-up compared with the other VitD status groups: the change in serum 25(OH)D was 34.9, 21.9, 4.5, -13.8 nmol/L from the lowest to the highest VitD status group (< 30, 30–50, 50–75, and > 75 nmol/L, respectively).

**Table 2:** 25(OH)D status of the study population

	31 years (*n* = 3650)	46 years (*n* = 3650)
25(OH)D, nmol/L	54.2 ± 18.5	64.8 ± 19.4
Prevalence*,* % (*n*)		
< 30 nmol/L	9.5 (346)	2.5 (92)
30–50 nmol/L	33.2 (1,213)	21.0 (765)
50–75 nmol/L	43.3 (1,581)	47.7 (1,740)
> 75 nmol/L	14.0 (510)	28.8 (1,053)

Values are displayed as mean (SD) or percentages with numbers in parentheses as % (*n*)

*25(OH)D* 25-hydroxyvitamin D

Figure 2 demonstrates the monthly variations in serum 25(OH)D concentrations at both timepoints. The mean seasonal difference in the serum 25(OH)D concentrations between the summer and winter months was 17.2 nmol/L at age 31: the mean in the winter months was 43.6 nmol/L (SD 15.4) and in the summer months 60.8 (SD 17.2). At age 46, this difference was reduced to 8.3 nmol/L: the mean in winter was 61.7 nmol/L (SD 19.7) and in summer, 69.0 nmol/L (SD 18.0). When comparing the 31- and 46-year data, we observed that the serum 25(OH)D concentrations increased by 41.6% in the winter months and 13.4% in the summer months during the 15-year follow-up period.

### Intake of VitD from diet and supplementation

Table 3 shows the contribution of dietary intake of VitD and the use of VitD supplements to serum 25(OH)D concentrations at age 46. The total VitD intake from diet, estimated from fortified dairy products, fortified fat spreads, and fish was on average 11.0 µg/d (SD 5.8), of which 6.0 µg/d came from fortified dairy products, 4.3 µg/d from fortified fat spreads, and 2.0 µg/d from fish. As a supplementary analysis, we tested for the differences in the intake of VitD between the sexes. The results are shown in the Online Resource 4. The estimated intake of VitD from the diet was higher in males than in females (12.3 µg vs. 10.1 µg, respectively, *P* < 0.001; Online Resource 4).

**Table 3:** Vitamin D intake from diet and the use of VitD supplements at 46 years and the differences by 25(OH)D cutoffs in nmol/L

	*n*	Mean ± SD	< 30	30–50	50–75	> 75	*P*
Total nutrition^a^, µg/d	3638	11.0 ± 5.8	8.9 ± 18.7	9.6 ± 5.1	11.4 ± 6.1	11.6 ± 5.8	< 0.001
Fortified dairy products^a^, µg/d	3555	6.0 ± 4.1	4.3 ± 3.9	5.0 ± 3.4	6.3 ± 4.4	6.6 ± 4.0	< 0.001
Fortified fat spreads^a^, µg/d	2766	4.3 ± 2.6	4.4 ± 2.7	4.2 ± 2.5	4.4 ± 2.8	4.1 ± 2.5	0.054
Fish^a^, µg/d	3384	2.0 ± 2.4	1.4 ± 2.0	1.8 ± 2.2	2.0 ± 2.4	2.2 ± 2.5	0.001
Use of supplements^b^, % (*n*)	3650						< 0.001
Regular		18.5 (672)	3.3 (3)	9.5 (73)	16.5 (288)	29.4 (310)	
Irregular		7.3 (268)	4.3 (4)	5.3 (40)	7.8 (135)	8.5 (89)	
No		74.2 (2,708)	92.4 (85)	85.2 (652)	75.7 (1,317)	62.1 (654)	
Supplementation dose^a^, µg/d, median (IQR)	866	10.0 (12.5)	4.4 (17.5)	10.0 (10)	10.0 (12.5)	16.3 (20.0)	< 0.001

Values are displayed as mean (SD) or percentages with numbers in parentheses as % (*n*), unless otherwise indicated

^a^Differences between the groups were tested using one-way analysis of variance for normally distributed and Kruskal–Wallis test for the non-parametric continuous variables

^b^Differences between the groups were tested using Pearson’s Chi-squared test for categorical variables

When comparing the intake of dietary VitD at 46 years, we observed differences by VitD status group: the intake of VitD from total nutrition (*P* < 0.001), fortified dairy products (*P* < 0.001), and fish (*P* < 0.027) was lowering towards the lowest VitD status group. There was no significant difference in VitD intake from fortified fat spreads between the VitD status groups. When the intakes of VitD from fortified dairy products and fat spreads were analyzed as quartiles, we observed a dose–response relationship between serum 25(OH)D concentrations and fortified dairy intake quartiles (60.4, 63.2, 65.0, and 68.8 nmol/L, *P* < 0.001, from the lowest to the highest quartile). Regarding the fortified fat spread intake quartiles, no evidence for a significant difference in VitD status was observed (66.1, 63.8, 64.0, and 64.9 nmol/L, *P* = 0.14, respectively).

VitD supplements were used by a quarter of the study population (25.8%) at age 46 (Table 3). Among this population, 71.5% used supplements regularly and 28.5% irregularly. Regular supplementation use was more common in the > 75 nmol/L (29.4%) and the 50–75 nmol/L (16.6%) groups compared with VitD status groups with 25(OH)D < 50 nmol/L (12.8%, *P* < 0.001). The frequency of regular use of VitD supplements in females was higher compared with males (24.5% vs*.* 10.8%, *P* < 0.001; Online Resource 4). The median dose of VitD supplementation in the study population at 46 years was 10.0 µg (IQR 12.5). The dose of VitD supplementation increased by the VitD status group (Table 3).

### Predictors of VitD status at 46 years

Figure 3 shows the results of the multivariable linear regression model, which was conducted to examine the independent association of multiple predictors of VitD status at 46 years. The 25(OH)D z-score (standardized for sex, season and latitude) at 46 years was positively predicted by the intake of fortified dairy products, fish intake, and regular use of VitD supplements. The reported intake of fortified fat spreads was not associated with the 46-year 25(OH)D z-score. The 46-year 25(OH)D *z* score was negatively associated with BMI, while the association with the 31-year 25(OH)D *z* score was positive. The coefficient of determination (*R*^2^) for the model was 0.16.

## Discussion

The present study represents one of the largest follow-up studies on VitD status from a longitudinal birth cohort and describes the effect of a national health policy among a middle-aged general population. According to our results, the population’s mean serum 25(OH)D concentration was improved by 10.6 nmol/L (SD 24.4) at 46 years (1997) compared to 31 years (2012–2013), likely reflecting the initiation of the food fortification policy in Finland.

We observed that the prevalence of serum 25(OH)D concentrations < 50 nmol/L decreased from 42.7 to 23.5% and < 30 nmol/L (indicating vitD deficiency [14]) from 9.5% to 2.5% during the 15 years of follow-up. The highest increase in serum 25(OH)D was found among those participants having the lowest VitD status at baseline, which is reflecting the effectiveness of the food fortification program. In addition, seasonal differences in mean 25(OH)D concentrations were reduced at the 46-year follow-up, and most of the study population maintained 25(OH)D concentration ≥ 50 nmol/L throughout the year. This follows the trend reported in a Finnish study based on Health 2000 and 2011 (H2000–2011) samples [18], which reported a decrease in the prevalence of 25(OH)D < 50 nmol/L from 55.7 to 9.1%. The difference in the prevalence of serum 25(OH)D < 50 nmol/L between these studies might be partly explained by a higher proportion of sampling conducted during the summer months in the H2011 survey compared with the NFBC1966 study, as well as with a higher percentage of the population from Northern Finland in our sample.

In contrast to the observations in Finland, another recent study from Northern Sweden found no clear time trend in serum 25(OH)D concentrations from 1986 to 2014 [19]. Relatively stable longitudinal 25(OH)D concentrations have also been described from Norway [20], the Longitudinal Aging Study Amsterdam [41], and three studies from the US [42–44], although notable seasonal variations were reported [20, 41]. To compare with other Nordic countries, the fortification policies have not been as systematic as in Finland, which might be explaining the differences in the change of VitD status (Online resource 1).

In addition to the national food fortification policy in Finland, many health information campaigns were communicating effectively about the beneficial effects of VitD. Nowadays, many people adhere to recommendations regarding VitD supplementation. In our sample, VitD supplementation was used by 26% of the population at 46 years, whereas the Finnish H2000–2011 study reported an increase from 11 to 41% [18]. Regular supplementation use had a significant effect on serum 25(OH)D concentration compared to no supplementation (an increase of 0.5 SD in regular supplement users vs. no supplement). It is noteworthy that in our questionnaire, the use of VitD supplementation was queried via an open-ended question [“In this section, write down the names, strengths and dosages of the medicines you are using. Do you use these medicines regularly or on a needs basis, and for which purpose? (on-the-shelf drugs, prescription drugs, ointments, vitamins and food supplements)”], which may have led to an underestimation of supplementation consumption. In addition, in the H2011 survey, the mean age of the population was higher (56 years), which may explain the difference we reported. Indeed, the use of supplements is usually more common in older age groups [18]. Our study population was mainly located in Northern Finland, whereas in the H2000–2011 study, the subjects were mainly from Southern Finland, suggesting possible differences in behavior and habits by region.

The mean intake of VitD from diet in our study population was 11.0 µg/d, which exceeds the Finnish and Nordic nutrition recommendations (10 µg/d) [14, 15]. This is a sign of a successful fortification policy. Sweden and Norway are also following fortification policies, but the amount of VitD added has been lower compared to Finland, and the VitD intake was lower in these countries in 2010–2011 (Online resource 1) [14]. In our study, dietary VitD was mainly obtained from milk products. We observed a dose–response effect in serum 25(OH)D concentrations across increasing milk intake quartiles. This finding is supported by earlier evidence [13]. A recent narrative review reported a positive association between the consumption of VitD fortified milk and VitD status [13]. In contrast, VitD from fortified fat spreads was not associated with serum 25(OH)D concentrations in our study, which was consistent with the H2000–2011 study [18].

The different effect of fortified milk products and fat spreads on serum 25(OH)D concentrations may be the result of health and lifestyle factors related to the use of these food items. In general, it has been suggested that the consumption of dairy products is associated with an overall healthy diet and a decreased risk for several diseases, especially cardiovascular diseases, type 2 diabetes, and metabolic syndrome [45, 46]. On the other hand, the consumption of fat spreads may be associated with unhealthier lifestyle and obesity [47], and our finding may reflect this difference. It can also be speculated that the difference arose, because the bioavailability of VitD is different between fortified milk and fat spreads [48, 49]. However, based on our study findings, we could dispute whether the current strategy of adding VitD in fat spreads is beneficial in increasing serum 25(OH)D concentrations in the general population. To the best of our knowledge, the current literature does not address this question.

In line with our study findings, there was no evidence in terms of a sex difference in VitD status according to a systematic review of observational studies published worldwide [50]. However, we found that males gained more VitD from their diet than females (12.3 vs. 10.1 µg, respectively), whereas females reported higher VitD supplementation than males (24.5% vs. 10.8%, respectively). This result was consistent with those of previous studies [15, 18, 51]. In general, energy intake from diet is higher in males [52], but it is noteworthy to acknowledge the difference in supplementation use.

We found the study population’s 31-year 25(OH)D to be predictive of their 25(OH)D at 46 years. To our knowledge, only one study has previously observed a similar finding in an adult population [20]. In our multivariable linear regression model, we were able to adjust for several confounders, including diet and supplementation. Our result may indicate that individual factors (e.g., sociodemographic, lifestyle, or genetics [53]) have an important role to play in VitD status in addition to nutrition and other VitD sources. This was shown in our findings as well: BMI, physical activity, smoking, and alcohol consumption differed between the VitD status groups, which is in line with previous knowledge from longitudinal studies [23, 31, 54, 55]. Individual biological pathways are also possibly linked to VitD status: absorption, liver and kidney function, chronic diseases, and medications. Future research is warranted to clarify these pathways and their role in individual VitD status.

Our study had multiple strengths. The study was based on a large general population-based cohort with a high participation rate and included a population from the same genetic, ethnic, and cultural backgrounds. As we were able to focus on specific age groups, possible confounding by age was accounted for [50]. Our findings also serve as important information for future studies investigating the effect of VitD food fortification on the risk for chronic diseases. In our analyses, we were able to control for several potential confounding factors that are known to be associated with serum 25(OH)D (e.g., BMI, season, and latitude) [23]. In addition, using a comprehensive questionnaire at 46 years, we were able to obtain information about the participants’ nutrition, and supplementation use.

One of the limitations of our study was that to respect of the timing of our study to assess the effect of fortification, it would have been more informative to use baseline serum 25(OH)D measurements before each wave of fortification. In addition, we had only one measurement per timepoint. A further limitation was that we did not have precise information about the participants’ supplementation use at age 31. Our supplementation data at 46 years was not concurrent with the nationwide report, but VitD supplementation was positively associated with serum 25(OH)D concentrations, so we consider the supplementation data reliable.

In conclusion, our results indicate a positive impact from the public health action on reducing the prevalence of serum 25(OH)D < 30 nmol/L and 50 nmol/L, and seasonal variations in VitD status among middle-aged adults in Finland. In addition to food fortification, regular VitD supplementation had an important effect on 25(OH)D, an increase of 0.5 SD compared to no supplementation. Although the fortification policy has influenced the entire population, we observed that the 31-year 25(OH)D concentrations in 1997 independently associated with serum 25(OH)D concentrations 15 years later in 2012–2013, which might be explained by behavioural or genetic factors. Our current study findings raise the question how to best locate the individuals who are at risk for serum 25(OH)D concentrations below 50 nmol/L and how to promote a further improvement of VitD status at population level.

## Supplementary Information

Below is the link to the electronic supplementary material.

Supplementary file1 (PDF 171 KB)

Supplementary file2 (PDF 122 KB)

Supplementary file3 (PDF 163 KB)

Supplementary file4 (PDF 117 KB)

## Data Availability

NFBC data are available from the University of Oulu, Infrastructure for Population Studies. Permission to use the data can be applied for research purposes via electronic material request portal. In the use of data, we follow the EU general data protection regulation (679/2016) and Finnish Data Protection Act. The use of personal data is based on cohort participant’s written informed consent at his/her latest follow-up study, which may cause limitations to its use. Please, contact NFBC project center (NFBCprojectcenter@oulu.fi) and visit the cohort website (www.oulu.fi/nfbc) for more information.

## Code availability

Code for the study is available from the corresponding author.
