# Supplementary material for: The determinants and longitudinal changes in vitamin D status in middle-age: a Northern Finland Birth Cohort 1966 study
Source: Eur J Nutr. 2021 Jun 17;60(8):4541–53. doi: 10.1007/s00394-021-02606-z (PMC8572212; doi:10.1007/s00394-021-02606-z)
Supplement: Supplementary file 1 — Supplementary file1 (PDF 171 KB) [file 394_2021_2606_MOESM1_ESM.pdf]

The determinants and longitudinal changes in vitamin D status in middle-age: A Northern Finland Birth Cohort 1966 study.

Helmi Ikonen[1]*, Johanna Lumme[2,3,4]*, Jussi Seppälä[1,5], Paula Pesonen[6], Terhi Piltonen[2,3,4], Marjo-Riitta Järvelin[1,7,8,9,10], Karl Heinz-Herzig[3,7,11], Jouko Miettunen[1,3], Maarit Niinimäki[2,3,4], Saranya Palaniswamy[1,8], Sylvain Sebert[1]†, Marja Ojaniemi[2,3,12]†

[1] Center for Life-Course Health Research, Faculty of Medicine, University of Oulu, 90014 Oulu, Finland
[2] PEDEGO Research Unit, University of Oulu, 90014 Oulu, Finland
[3] Medical Research Center Oulu, Oulu University Hospital and University of Oulu, 90014 Oulu, Finland
[4] Department of Obstetrics and Gynecology, Oulu University Hospital, 90220 Oulu, Finland
[5] Department of Mental and Substance Use Disorders, South Carelia Social and Healthcare District, Lappeenranta, Finland
[6] Infrastructure for Population Studies, Faculty of Medicine, University of Oulu, 90014 Oulu, Finland
[7] Biocenter Oulu, University of Oulu, 90014 Oulu, Finland
[8] Department of Epidemiology and Biostatistics, MRC Centre for Environment and Health, School of Public Health, Imperial College, London W2 1PG, UK
[9] Department of Life Sciences, College of Health and Life Sciences, Brunel University London, Kingston Lane, Uxbridge, Middlesex UB8 3PH, UK
[10] Unit of Primary Care, Oulu University Hospital, Oulu, Finland
[11] Institute of Biomedicine, Medical Research Center, University of Oulu, 90014 Oulu, Finland
[12] Department of Pediatrics and Adolescence, Oulu University Hospital, 90220 Oulu, Finland

Equal contribution *, equal contribution †

Address correspondence to: Sylvain Sebert, University of Oulu Center for Life Course Health Research Aapistie 5 B, Fin-90220 Oulu, Finland. Telephone: +358294488004. Email: sylvain.sebert@oulu.fi. ORCID: 0000-0001-6681-6983.

Electronic Supplementary Material
Online resource 1

Table 1. Vitamin D fortification in fluid milk products and fat spreads in Nordic countries.

**Product**	**Finland**[1, 2]	**Sweden**[1, 3, 4]	**Norway**[5]	**Denmark**[6, 7]	**Iceland**[1]
**Fluid milk products**	*2002: 0.5ug/100g*<br><br>*2010: 1 µg /100 g fluid milk products, lactose-free and vegetable-based alternatives, yoghurt, sourmilk*	*2007: extra low-fat milk (<1.5% fat) 0.38-0.5 µg /g*<br><br>*2018: milk, fermented milk (≤3% fat), including lactose-free and vegetable-based alternatives 0.75-1.1 µg/100 g*	*2006: Extra low fat and lactose free milk, 0.4 µg /100 g*	*No systematic fortification*	*No systematic fortification*
**Fat spread**	*2002: 10ug/100g*<br>*2010: 20ug/100g*	*2007: margarine and cooking fats 7.5-10 µg/100 g*<br>*2018: 19.5-21.0 µg/100 g*	*2006: 10 µg /100 g*	*No systematic fortification*	*No systematic fortification*
**Other**				*Fortification allowed since 2005 in fat spreads, sports drinks and lactose-free milk products*	*Some milk products, some domestic foods (most fat spreads) and some imported foods (vegetable oils, cereals) are fortified.*

References:

1. Itkonen ST, Andersen R, Björk AK, et al (2020) Vitamin D status and current policies to achieve adequate vitamin D intake in the Nordic countries. Scand J Public Health 140349481989687. https://doi.org/10.1177/1403494819896878

2. Itkonen S, Erkkola M, Lamberg-Allardt C (2018) Vitamin D Fortification of Fluid Milk Products and Their Contribution to Vitamin D Intake and Vitamin D Status in Observational Studies—A Review. Nutrients 10:1054. https://doi.org/10.3390/nu10081054

3. Summerhays E, Eliasson M, Lundqvist R, et al (2019) Time trends of vitamin D concentrations in northern Sweden between 1986 and 2014: a population-based cross-sectional study. Eur J Nutr. https://doi.org/10.1007/s00394-019-02142-x

4. Livsmedelsverkets författningssamling, 2018. https://www.livsmedelsverket.se/globalassets/om-oss/lagstiftning/berikn---kosttillsk---livsm-spec-gr-fsmp/livsfs-2018-5_web.pdf. Accessed 10 Mar 2021

5. Vitamin D i Norge: Behov for tiltak for å sikre god vitamin D-status? 2018.

https://www.helsedirektoratet.no/rapporter/vitamin-d-i-norge-behov-for-tiltak-for-a-sikre-god-vitamin-d-status/Vitamin D i Norge – Behov for tiltak for å sikre god vitamin D-status.pdf/_/attachment/inline/b307f785-c4cc-4fde-aec1-ebc86fdd0b4f:829f3ad84cbdf. Accessed 10 Mar 2021

6. Grønborg IM, Tetens I, Ege M, et al (2019) Modelling of adequate and safe vitamin D intake in Danish women using different fortification and supplementation scenarios to inform fortification policies. Eur J Nutr 58:227–232. https://doi.org/10.1007/s00394-017-1586-9

7. Grønborg IM, Tetens I, Christensen T, et al (2020) Vitamin D-fortified foods improve wintertime vitamin D status in women of Danish and Pakistani origin living in Denmark: a randomized controlled trial. Eur J Nutr 59:741–753. https://doi.org/10.1007/s00394-019-01941-6
