# Supplementary material for: The determinants and longitudinal changes in vitamin D status in middle-age: a Northern Finland Birth Cohort 1966 study
Source: Eur J Nutr. 2021 Jun 17;60(8):4541–53. doi: 10.1007/s00394-021-02606-z (PMC8572212; doi:10.1007/s00394-021-02606-z)
Supplement: Supplementary file 2 — Supplementary file2 (PDF 122 KB) [file 394_2021_2606_MOESM2_ESM.pdf]

The determinants and longitudinal changes in vitamin D status in middle-age: A Northern Finland Birth Cohort 1966 study.

Helmi Ikonen[1]*, Johanna Lumme[2,3,4]*, Jussi Seppälä[1,5], Paula Pesonen[6], Terhi Piltonen[2,3,4], Marjo-Riitta Järvelin[1,7,8,9,10], Karl Heinz-Herzig[3,7,11], Jouko Miettunen[1,3], Maarit Niinimäki[2,3,4], Saranya Palaniswamy[1,8], Sylvain Sebert[1]†, Marja Ojaniemi[2,3,12]†

[1] Center for Life-Course Health Research, Faculty of Medicine, University of Oulu, 90014 Oulu, Finland
[2] PEDEGO Research Unit, University of Oulu, 90014 Oulu, Finland
[3] Medical Research Center Oulu, Oulu University Hospital and University of Oulu, 90014 Oulu, Finland
[4] Department of Obstetrics and Gynecology, Oulu University Hospital, 90220 Oulu, Finland
[5] Department of Mental and Substance Use Disorders, South Carelia Social and Healthcare District, Lappeenranta, Finland
[6] Infrastructure for Population Studies, Faculty of Medicine, University of Oulu, 90014 Oulu, Finland
[7] Biocenter Oulu, University of Oulu, 90014 Oulu, Finland
[8] Department of Epidemiology and Biostatistics, MRC Centre for Environment and Health, School of Public Health, Imperial College, London W2 1PG, UK
[9] Department of Life Sciences, College of Health and Life Sciences, Brunel University London, Kingston Lane, Uxbridge, Middlesex UB8 3PH, UK
[10] Unit of Primary Care, Oulu University Hospital, Oulu, Finland
[11] Institute of Biomedicine, Medical Research Center, University of Oulu, 90014 Oulu, Finland
[12] Department of Pediatrics and Adolescence, Oulu University Hospital, 90220 Oulu, Finland

Equal contribution *, equal contribution †

Address correspondence to: Sylvain Sebert, University of Oulu Center for Life Course Health Research Aapistie 5 B, Fin-90220 Oulu, Finland. Telephone: +358294488004. Email: sylvain.sebert@oulu.fi. ORCID: 0000-0001-6681-6983.

Electronic Supplementary Material

Online resource 2

Fig.1 Sensitivity analysis with inclusion of outlier data (*n*=112)

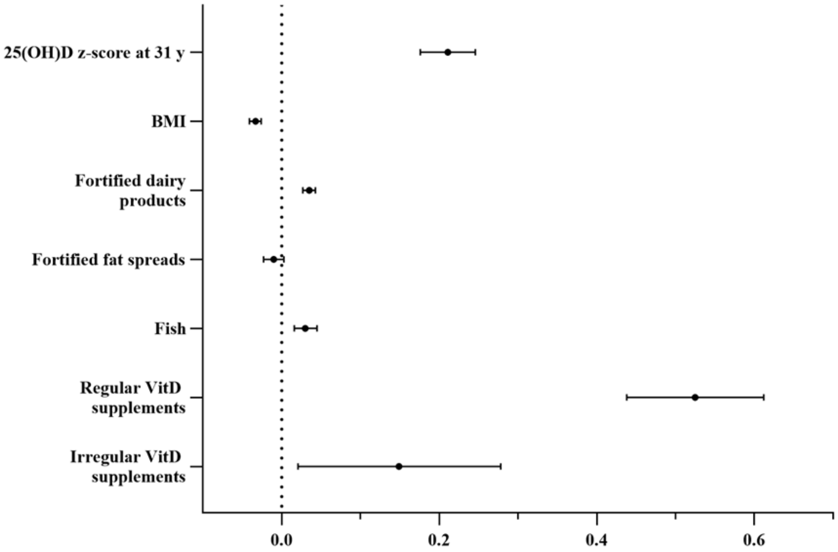

β Coefficient	95% Confidence Interval	*P*
0.25	0.18, 0.21	<0.001
-0.026	-0.041, -0.033	<0.001
0.035	0.027, 0.043	<0.001
-0.01	-0.023, 0.003	0.14
0.030	0.016, 0.045	<0.001
0.53	0.44, 0.61	<0.001
0.15	0.021, 0.28	0.023

For VitD supplementation, no VitD supplementation was used as a reference category.

Fig.2 Sensitivity analysis with exclusion of oral contraceptive and hormonal replacement therapy users (*n*=159)

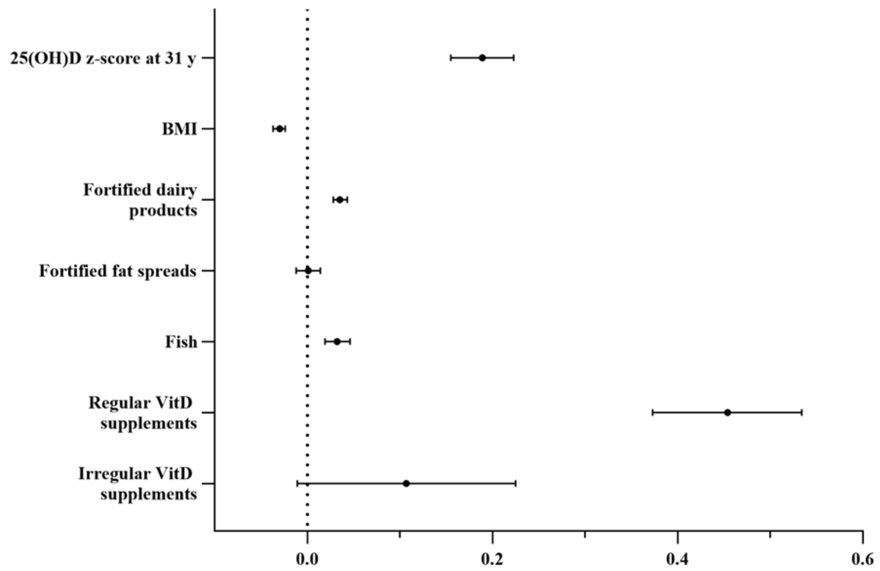

β Coefficient	95% Confidence Interval	*P*
0.19	0.16, 0.22	<0.001
-0.03	-0.037, -0.024	<0.001
0.035	0.028, 0.043	<0.001
0.001	-0.012, 0.014	0.88
0.032	0.019, 0.046	<0.001
0.45	0.37, 0.53	<0.001
0.11	-0.011, 0.23	0.075

For VitD supplementation, no VitD supplementation was used as a reference category.
