# Supplementary material for: The determinants and longitudinal changes in vitamin D status in middle-age: a Northern Finland Birth Cohort 1966 study
Source: Eur J Nutr. 2021 Jun 17;60(8):4541–53. doi: 10.1007/s00394-021-02606-z (PMC8572212; doi:10.1007/s00394-021-02606-z)
Supplement: Supplementary file 3 — Supplementary file3 (PDF 163 KB) [file 394_2021_2606_MOESM3_ESM.pdf]

The determinants and longitudinal changes in vitamin D status in middle-age: A Northern Finland Birth Cohort 1966 study.

Helmi Ikonen[1]*, Johanna Lumme[2,3,4]*, Jussi Seppälä[1,5], Paula Pesonen[6], Terhi Piltonen[2,3,4], Marjo-Riitta Järvelin[1,7,8,9,10], Karl Heinz-Herzig[3,7,11], Jouko Miettunen[1,3], Maarit Niinimäki[2,3,4], Saranya Palaniswamy[1,8], Sylvain Sebert[1]†, Marja Ojaniemi[2,3,12]†

[1] Center for Life-Course Health Research, Faculty of Medicine, University of Oulu, 90014 Oulu, Finland
[2] PEDEGO Research Unit, University of Oulu, 90014 Oulu, Finland
[3] Medical Research Center Oulu, Oulu University Hospital and University of Oulu, 90014 Oulu, Finland
[4] Department of Obstetrics and Gynecology, Oulu University Hospital, 90220 Oulu, Finland
[5] Department of Mental and Substance Use Disorders, South Carelia Social and Healthcare District, Lappeenranta, Finland
[6] Infrastructure for Population Studies, Faculty of Medicine, University of Oulu, 90014 Oulu, Finland
[7] Biocenter Oulu, University of Oulu, 90014 Oulu, Finland
[8] Department of Epidemiology and Biostatistics, MRC Centre for Environment and Health, School of Public Health, Imperial College, London W2 1PG, UK
[9] Department of Life Sciences, College of Health and Life Sciences, Brunel University London, Kingston Lane, Uxbridge, Middlesex UB8 3PH, UK
[10] Unit of Primary Care, Oulu University Hospital, Oulu, Finland
[11] Institute of Biomedicine, Medical Research Center, University of Oulu, 90014 Oulu, Finland
[12] Department of Pediatrics and Adolescence, Oulu University Hospital, 90220 Oulu, Finland

Equal contribution *, equal contribution †

Address correspondence to: Sylvain Sebert, University of Oulu Center for Life Course Health Research Aapistie 5 B, Fin-90220 Oulu, Finland. Telephone: +358294488004. Email: sylvain.sebert@oulu.fi. ORCID: 0000-0001-6681-6983.

Electronic Supplementary Material
Online resource 3

Table 1. Comparison of the total sample with serum 25(OH)D measurements and the sample with repeated serum 25(OH)D measurements[1].

	**31 years**		**46 years**	
	**Total (*n* = 5,383-5,571)**	**Repeated (*n* = 3,538-3,650)**	**Total (*n* = 5,319-5,659)**	**Repeated (*n* = 3,467-3,650)**
**Females, % (*n*)**	51.9 (2,893)	56.2 (2,051)	55.8 (3,156)	56.2 (2,051)
**Marital status, % (*n*)**				
Married	72.7 (4,004)	75.0 (2,710)	78.8 (4,230)	78.9 (2,752)
Unmarried	27.3 (1,503)	25.0 (905)	21.2 (1,136)	21.1 (738)
**Occupational status, % (*n*)**				
Higher level employee	17.6 (946)	18.6 (660)	19.1 (1,019)	17.9 (619)
Lower level employee/entrepreneur	38.6 (2,079)	41.0 (1,452)	28.4 (1,517)	28.4 (984)
Manual worker/farmer	29.7 (1,599)	27.7 (979)	46.9 (2,503)	48.7 (1,688)
Not working	14.2 (765)	12.7 (449)	5.5 (296)	5.1 (176)
**BMI, kg/m^2, mean (SD)**	24.7 ± 4.2	24.5 ± 4.0	26.9 ± 4.9	26.8 ± 4.8
**Physical activity, MET hours/week[2], median (IQR)**	10.9 (16.9)	11.3 (16.6)	13.1 (19.0)	13.1 (17.9)
**Smoking, % (*n*)**				
Non-smoker	44.8 (2,458)	48.8 (1,760)	53.9 (2,865)	54.7 (1,892)
Former smoker	25.5 (1,400)	26.5 (956)	27.1 (1,439)	27.3 (946)
Current smoker	29.6 (1,624)	24.6 (888)	19.1 (1,015)	28.0 (624)
**Alcohol consumption[3], % (*n*)**				
Abstainer	9.7 (523)	9.7 (344)	10.3 (553)	10.4 (365)
Low-risk drinker	84.7 (4,557)	85.4 (3,022)	81.3 (4,373)	81.8 (2,864)
High-risk drinker	5.6 (303)	4.9 (172)	8.4 (452)	7.7 (271)
**Season of the blood sampling[4], % (*n*)**				
High Vit D season	61.9 (3,416)	61.5 (2,229)	41.5 (2,350)	42.0 (1,532)
Low Vit D season	38.1 (2,104)	38.5 (1,396)	58.5 (3,309)	58.0 (2,118)
**Latitude[5], % (*n*)**				
60°N	15.5 (861)	15.0 (547)	32.0 (1,809)	19.5 (712)
65°N	19.4 (1,082)	20.8 (758)	24.4 (1,381)	28.7 (1,048)
≥65°N	65.1 (3,628)	64.2 (2,345)	43.6 (2,469)	51.8 (1,890)

[1]Values are displayed as percentages with numbers in parentheses as % (*n*) or mean (SD), unless otherwise indicated.

[2]The MET physical activity scores in hours per week (frequency and duration of leisure time activities).

[3]Alcohol intake: abstainer (0 g/d), low-risk drinker (males ≤40 g/d, females ≤20 g/d), and high-risk drinker (males >40 g/d, females >20 g/d).

[4]High Vit D season: summer (1 June–30 August) and autumn (1 September–31 October). Low vitamin D season: winter (1 November–31 March) and spring (1 April–31 May).

[5]Latitude: 60°N, Helsinki and surrounding areas; 65°N, the city of Oulu; >65°N, the northernmost provinces of Oulu and Lapland.
