# Supplementary material for: The determinants and longitudinal changes in vitamin D status in middle-age: a Northern Finland Birth Cohort 1966 study
Source: Eur J Nutr. 2021 Jun 17;60(8):4541–53. doi: 10.1007/s00394-021-02606-z (PMC8572212; doi:10.1007/s00394-021-02606-z)
Supplement: Supplementary file 4 — Supplementary file4 (PDF 117 KB) [file 394_2021_2606_MOESM4_ESM.pdf]

The determinants and longitudinal changes in vitamin D status in middle-age: A Northern Finland Birth Cohort 1966 study.

Helmi Ikonen[1]*, Johanna Lumme[2,3,4]*, Jussi Seppälä[1,5], Paula Pesonen[6], Terhi Piltonen[2,3,4], Marjo-Riitta Järvelin[1,7,8,9,10], Karl Heinz-Herzig[3,7,11], Jouko Miettunen[1,3], Maarit Niinimäki[2,3,4], Saranya Palaniswamy[1,8], Sylvain Sebert[1]†, Marja Ojaniemi[2,3,12]†

[1] Center for Life-Course Health Research, Faculty of Medicine, University of Oulu, 90014 Oulu, Finland
[2] PEDEGO Research Unit, University of Oulu, 90014 Oulu, Finland
[3] Medical Research Center Oulu, Oulu University Hospital and University of Oulu, 90014 Oulu, Finland
[4] Department of Obstetrics and Gynecology, Oulu University Hospital, 90220 Oulu, Finland
[5] Department of Mental and Substance Use Disorders, South Carelia Social and Healthcare District, Lappeenranta, Finland
[6] Infrastructure for Population Studies, Faculty of Medicine, University of Oulu, 90014 Oulu, Finland
[7] Biocenter Oulu, University of Oulu, 90014 Oulu, Finland
[8] Department of Epidemiology and Biostatistics, MRC Centre for Environment and Health, School of Public Health, Imperial College, London W2 1PG, UK
[9] Department of Life Sciences, College of Health and Life Sciences, Brunel University London, Kingston Lane, Uxbridge, Middlesex UB8 3PH, UK
[10] Unit of Primary Care, Oulu University Hospital, Oulu, Finland
[11] Institute of Biomedicine, Medical Research Center, University of Oulu, 90014 Oulu, Finland
[12] Department of Pediatrics and Adolescence, Oulu University Hospital, 90220 Oulu, Finland

Equal contribution *, equal contribution †

Address correspondence to: Sylvain Sebert, University of Oulu Center for Life Course Health Research Aapistie 5 B, Fin-90220 Oulu, Finland. Telephone: +358294488004. Email: sylvain.sebert@oulu.fi. ORCID: 0000-0001-6681-6983.

Electronic Supplementary Material
Online resource 4

Table 1. Comparisons of serum 25(OH)D concentrations and intake of vitamin D from diet and supplements between the female and male participants in the Northern Finland Birth Cohort 1966.[1]

	n	**Female**	***n***	**Male**	***P***
**25(OH)D at age 31**[2], nmol/L	2,051	43.9 ± 18.5	1,599	54.5 ± 18.5	0.39
**25(OH)D at age 46**[2], nmol/L	2,051	65.2 ± 19.4	1,599	64.1 ± 19.3	0.083
**Dietary intake of vitamin D at age 46**					
**Total nutrition**[2], µg/d	2,046	10.1 ± 5.1	1,592	12.3 ± 6.5	<0.001
**Dairy products**[2], µg/d	2,006	5.6 ± 3.4	1,549	6.6 ± 4.8	<0.001
**Fat spreads**[2], µg/d	1,550	3.7 ± 2.4	1,216	5.1 ± 2.7	<0.001
**Fish**[2]**,** µg/d	1,922	1.9 ± 2.2	1,462	2.1 ± 2.6	0.077
**Use of supplements**[3]**,** % (*n*)	2,051		1,599		<0.001
Regular		24.5 (502)		10.8 (172)	
Irregular		9.9 (204)		4.0 (64)	
No		65.6 (1,345)		85.2 (1,363)	
**Supplementation dose**[2], µg/d, median (IQR)	643	11.3 (12.5)	223	10.0 (15.0)	0.009

[1]Values are displayed as percentages with numbers in parentheses as % (*n*) or mean (SD), unless otherwise indicated.

[2]The differences between the sexes were tested using independent samples *t*-tests for normally distributed and the Mann–Whitney *U* test for non-parametric continuous variables.

[3]The differences between the sexes were tested using the Pearson's chi-squared test for categorical variables.
